# Supplementary material for: Electric-Field-Induced Phase Transformation and Frequency-Dependent Behavior of Bismuth Sodium Titanate–Barium Titanate
Source: Materials (Basel). 2020 Feb 27;13(5):1054. doi: 10.3390/ma13051054 (PMC7084422; doi:10.3390/ma13051054)
Supplement: Supplementary file 1 [file materials-13-01054-s001.pdf]

materials

Supplementary Materials

Electric-Field-Induced Phase Transformation and Frequency-Dependent Behaviour of Bismuth Sodium Titanate–Barium Titanate

Kai-Yang Lee[1], Xi Shi[2], Nitish Kumar[2], Mark Hoffman[2], Martin Etter[3], Stefano Checchia[4,5], Jens Winter[6], Lucas Lemos da Silva[1], Daniela Seifert[1] and Manuel Hinterstein[1,2].

[1]*Institute for Applied Materials, Karlsruhe Institute of Technology, 76131 Karlsruhe, Germany.*

[2]*School of Materials Science and Engineering, UNSW Sydney, 2052 Sydney, Australia.*

[3]*Deutsches Elektronensynchrotron DESY, 22607 Hamburg, Germany.*

[4]*European Synchrotron Radiation Facility ESRF, 38043 Grenoble, France.*

[5]*MAX IV Laboratory, Lund University, 22100 Lund, Sweden.*

[6]*Department of Physics, University of Siegen, 57068 Siegen, Germany.*

The electric field response of the lead-free solid solution (1-x)$Bi_{0.53}Na_{0.47}TiO_3$–$x$$BaTiO_3$ (BNT–BT) in the higher BT composition range with x = 0.12 was investigated using *in situ* synchrotron X-ray powder diffraction. An introduced Bi-excess non-stoichiometry caused an extended morphotropic phase boundary, leading to an unexpected fully reversible relaxor to ferroelectric (R–FE) phase transformation behaviour. By varying the field frequency in a broad range from 10^{-4} up to 10^{2} Hz, BNT–12BT showed a frequency-dependent gradual suppression of the field induced ferroelectric phase transformation in favour of the relaxor state. A frequency triggered self-heating within the sample was found and the temperature increase exponentially correlated with the field frequency. The effects of a lowered phase transformation temperature $T_{R–FE}$, caused by the non-stoichiometric composition, were observed in the experimental setup of the freestanding sample. This frequency-dependent investigation of an R–FE phase transformation is unlike previous macroscopic studies, in which heat dissipating metal contacts are used.

This supplementary material section provides descriptions and details about the experimental sample environment, the data diffraction methods and information about the Rietveld refinement process.

Sample environment

Figure S1 shows a photograph of the sample environment used at beamline P02.1 at the *Deutsches Elektronen-Synchrotron* (DESY) in Hamburg. The sample environment was specifically designed in order to apply high electric fields in transmission geometry with the possibility of varying the sample orientation. The sample environment is mounted on the sample environment circle ω of the goniometer. This circle is concentric with the 2θ circle of the MAD. The bar shaped sample is mounted on a Teflon sample holder and electrically

connected with two opposing metal wires. With these wires the high voltage is applied. In order to apply high voltages, a Polyimide tube is mounted on the Teflon sample holder and filled with silicon oil. The end is sealed with modelling clay to prevent spilling. An acrylic resin hood is mounted for safety reasons to shield the sample and high voltage environment. As depicted in Figure S2, for experiments with a 2D detector only a single sample orientation is necessary. In this case the electric field vector is perpendicular to the incoming beam. However, when a high resolution MAD is used, the sample orientation can be varied by rotating the ω circle.

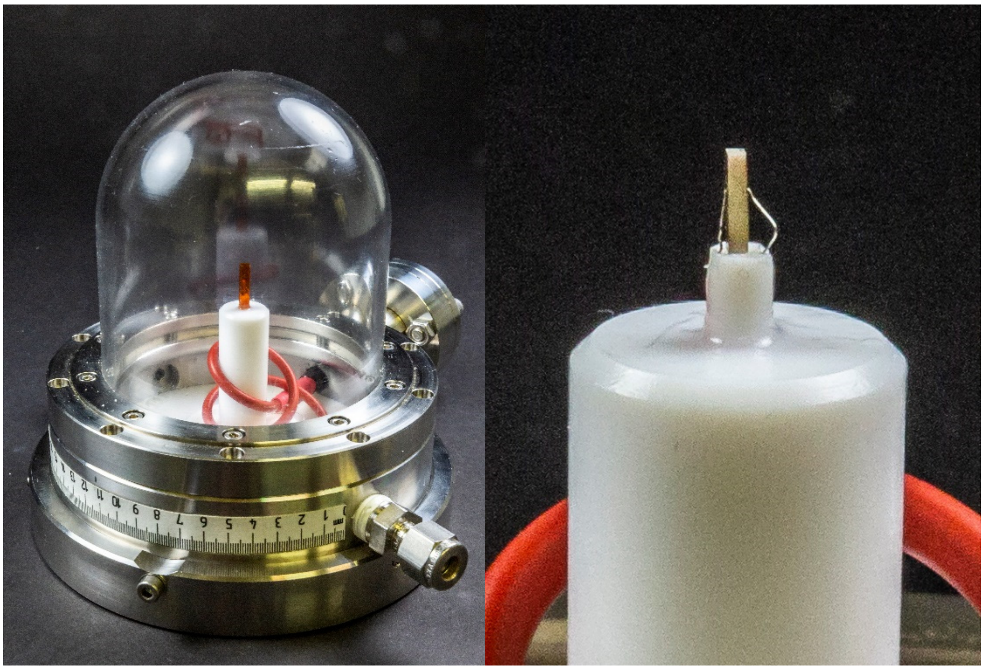

Figure S1: Entire sample environment with glass protection (left). Rectangular sample on sample holder, pinned by wires on both electrode sides (right).

2D detector diffraction data collection

Figure S2 shows different setups for a 2D data acquisition. When e.g. the first 10 reflections should be measured, the 2D detector has to be placed in a certain distance (Figure S2a). The same information can be obtained when the 2D detector is not placed with the direct beam in the centre, but in one of the corners (Figure S2b). This way, the Q-range is the same, but the angular resolution is twice as high. Figure S2c shows the setup with the direct beam in the centre for the same distance. Here full orientation information can be obtained for only three reflections. Since the sample-to-detector distance is equal in b and c, the angular resolution remains unchanged.

Since the electric field is unidirectional, the induced response of the sample is symmetric with respect to the (vertical) field vector direction, which means the 90° section covered by the 2D detector in Figure S2b is sufficient for data analysis. All texture effects are visible and the three remaining quadrants yield redundant information. To therefore gain a high Q-range and simultaneously maintain a high angular resolution, the setup in Figure S2b is chosen. For more details of the experimental setup, other works can be considered [1–3].

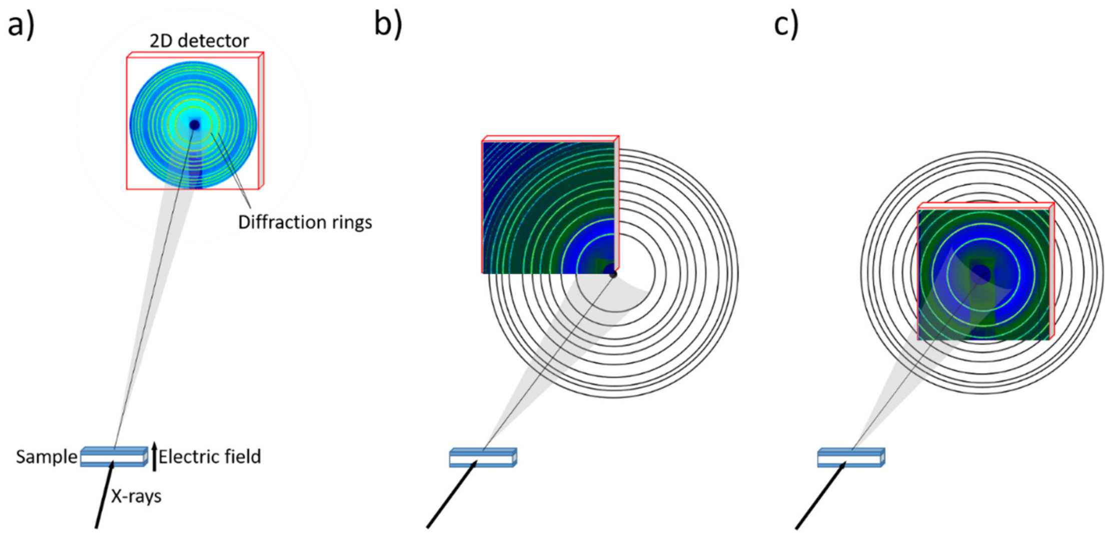

Figure S2: Schematic experimental setup of in situ diffraction measurements in transmission geometry using a 2D detector. Comparison of data acquisition of a 360° image with a high Q-range using a higher sample-to-detector distance (a), a 90° section (one quadrant, b) and a 360° diffraction image with a low Q-range (all quadrants, c).

Stroboscopic mode of data collection

Using stroboscopy, a periodic waveform is applied to the sample and divided into time slots by a field-programmable gate-array (FPGA) [4]. A waveform generator served to set the desired waveform, amplitude and frequency of the electric field. Since the electric field surges are triggered by the waveform generator, the intensity counts can be sorted by the different times passed after the triggering signal. Thus, the intensity for every time bin with the same time passed can be summed up regardless of the number of cycles.

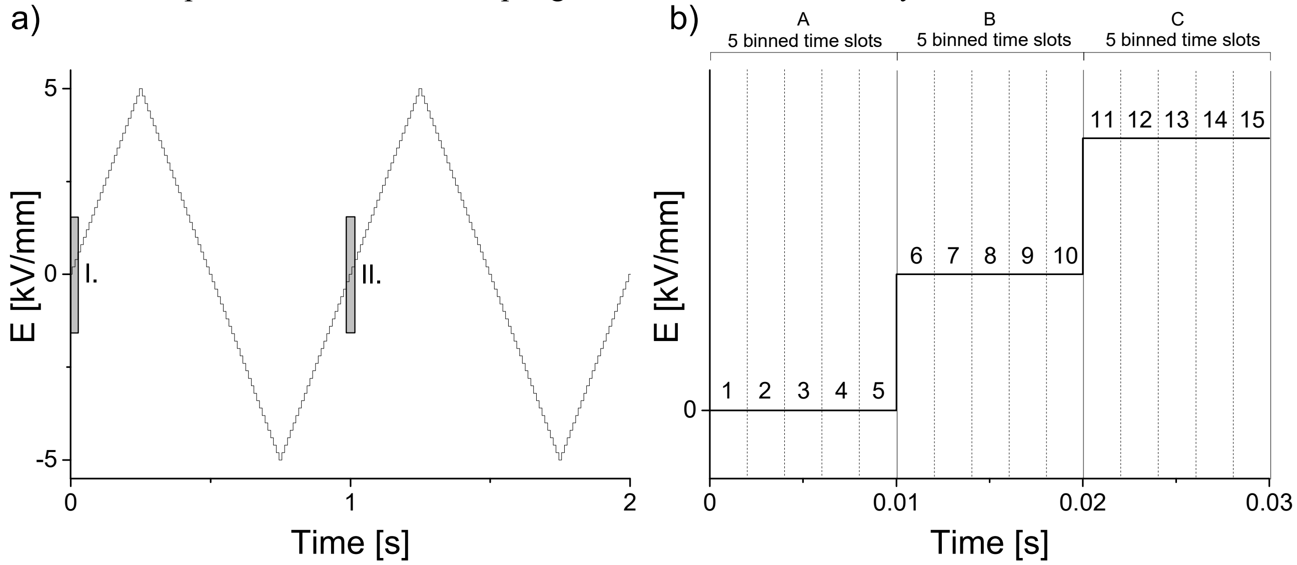

Figure S3: An exemplary electric field cycling illustrates the stroboscopic measuring mode. Two applied field cycles with grey marked squares (a) consist of 15 time slots, grouped to three time bins (A, B, C) with five time slots (b).

Figure S3a shows two cycles of a triangular waveform with rectangular steps and an amplitude of 5 kV/mm. Zooming into the grey marked square I. (Figure S3b), 15 time slots are differentiated using that specific time resolution. Each five time slots have the same electric field value due to the stepwise wave function. Therefore, these five time slots are

binned into A, B and C, respectively. The grey marked square II of the second field cycle possesses the same field values, so that the intensities of all A bins are summed up to a total value A. Likewise, bins B and C of cycle I and II are added up to total values of B and C. In this way, not only two, but also any number of cycles of A–C can be added up, so that the total intensity values increase with every cycle step. A–C in the marked area represent only a fraction of a full cycle, therefore further bins (D, E, F, …) must be taken into account for a complete cycle. By this method, one total summed up field cycle with sufficient statistics results, which would otherwise not be technically possible.

Refinement of temperature-dependent data

The measured diffraction patterns of the temperature series was refined using the Rietveld method via the software package Material Analysis Using Diffraction (MAUD) [5]. Figure S4 shows the measured diffraction and calculated refined data at T = 125 °C, the maximum temperature of the series. A structure model with a cubic *Pm*-3*m* phase was selected to refine the pseudo-cubic relaxor state, therefore neglecting a minor unit cell distortion, which causes the asymmetry shown in the inset of Figure S4.

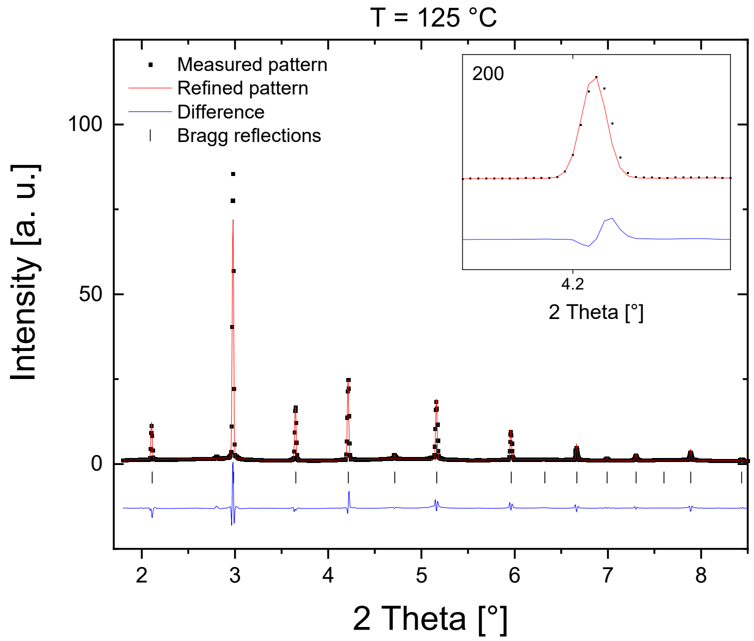

Figure S4: The measured diffraction data (black dots), the calculated Rietveld refined pattern (red) and the difference plot (blue) for the last step at 125 °C of the temperature series measurement. The inset shows the 200_C reflection.

The diffraction pattern was first refined and the results passed to the next temperature step as input parameters. This successive refinement process was repeated for all temperature diffraction data. During this procedure, the refined parameters were as follows: the general background level, the overall scale factor, the cubic lattice parameter, the Popa rules parameter to account for microstrains [6] and the isotropic Debye-Waller factors. The latter were refined separately for all A-site atoms constraint, titanium and oxygen atoms.

Electric field cycling of the sample

In order to verify the reversibility of the phase transformation behaviour of BNT–12BT, the sample was cycled with an electric field with an amplitude of 5 kV/mm. The first 10^2 cycles were applied with a frequency of 0.1 Hz, the subsequent 10^3 cycles with 1 Hz and other 10^4

cycles with 10 Hz. Figure S5 shows the 200_C reflections at remanent field state (a) and at maximum field values (b). The preserved characteristics of the reflections indicate a full reversible field-dependent phase behaviour. The minor decrease in intensity suggests fatigue effects.

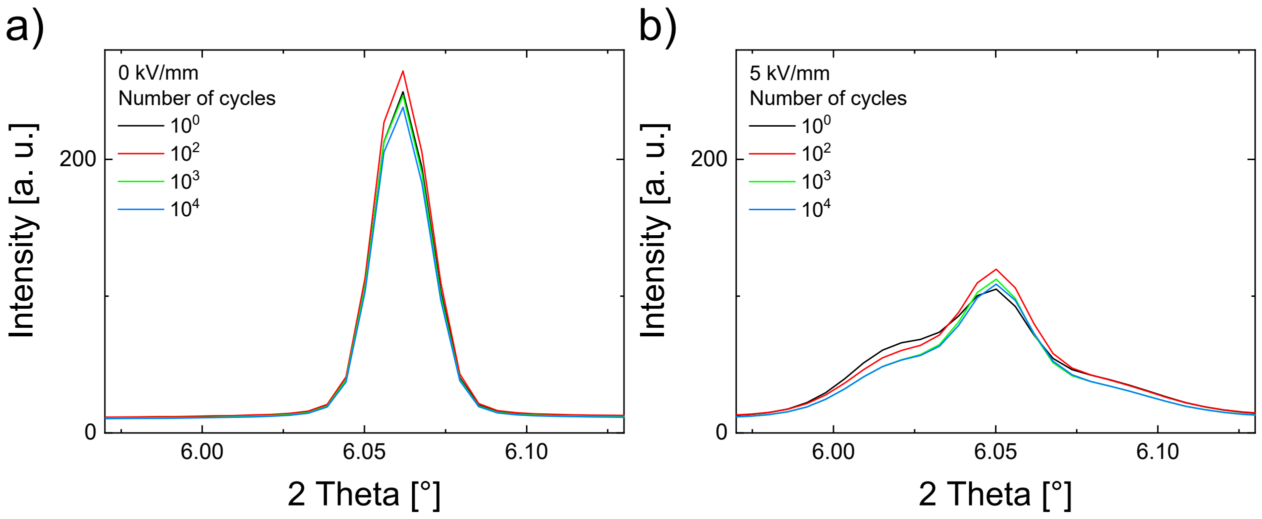

Figure S5: The 200_C reflections at 0 kV/mm (a) and at 5 kV/mm (b) of the unpoled state (black line) and after increasing number of electric field cycles (coloured lines).

1. Daniels JE, Pramanick A, Jones JL. Time-resolved characterization of ferroelectrics using high-energy X-ray diffraction. *IEEE Trans Ultrason Ferroelectr Freq Control*. 2009;56(8):1539-1545. doi:10.1109/TUFFC.2009.1218

2. Daniels JE. Determination of directionally dependent structural and microstructural information using high-energy X-ray diffraction. *J Appl Crystallogr*. 2008;41(6):1109-1114. doi:10.1107/s0021889808031488

3. Pramanick A, Damjanovic D, Daniels JE, Nino JC, Jones JL. Origins of electro-mechanical coupling in polycrystalline ferroelectrics during subcoercive electrical loading. *J Am Ceram Soc*. 2011;94(2):293-309. doi:10.1111/j.1551-2916.2010.04240.x

4. Choe H, Gorfman S, Hinterstein M, et al. Combining high time and angular resolutions: time-resolved X-ray powder diffraction using a multi-channel analyser detector. *J Appl Crystallogr*. 2015;48(3):970-974. doi:10.1107/S1600576715004598

5. Matthies S, Lutterotti L, Wenk HR. Advances in Texture Analysis from Diffraction Spectra. *J Appl Crystallogr*. 1997;30(1):31-42. doi:10.1107/S0021889896006851

6. Popa NC. The (hkl) Dependence of Diffraction-Line Broadening Caused by Strain and Size for all Laue Groups in Rietveld Refinement. *J Appl Crystallogr*. 1998;31(2):176-180. doi:10.1107/S0021889897009795

© 2020 by the authors. Licensee MDPI, Basel, Switzerland. This article is an open access article distributed under the terms and conditions of the Creative Commons Attribution (CC BY) license (http://creativecommons.org/licenses/by/4.0/).
